# Case Report: Primary Leiomyosarcoma of the breast with unusual metastasis to the femur

**DOI:** 10.12688/f1000research.5213.1

**Published:** 2014-09-04

**Authors:** Evgeniya Sokolovskaya, Zheng Liu, Kelly Weintraub, Arpad Szallasi, Yasmeen Shariff

**Affiliations:** 1 Department of Radiology, Monmouth Medical Center, Long Branch, USA; 2 Department of Pathology, Monmouth Medical Center, Long Branch, USA

## Abstract

With less than 40 cases reported, primary leiomyosarcoma is an extremely rare form of breast cancer (less than 0.0006% of cases) with unpredictable biological behavior that usually presents as a slow growing, mobile mass in middle age women. Most cases are low-grade and are cured by complete excision with wide margins. After surgical resection, late local recurrence and distant hematogenous metastasis to lungs and liver is, however, well-documented. To the best of our knowledge, bone metastasis has never been reported. Here we present a case of primary leiomyosarcoma of the breast metastatic to the femur.

## Case report

**Figure 1.**
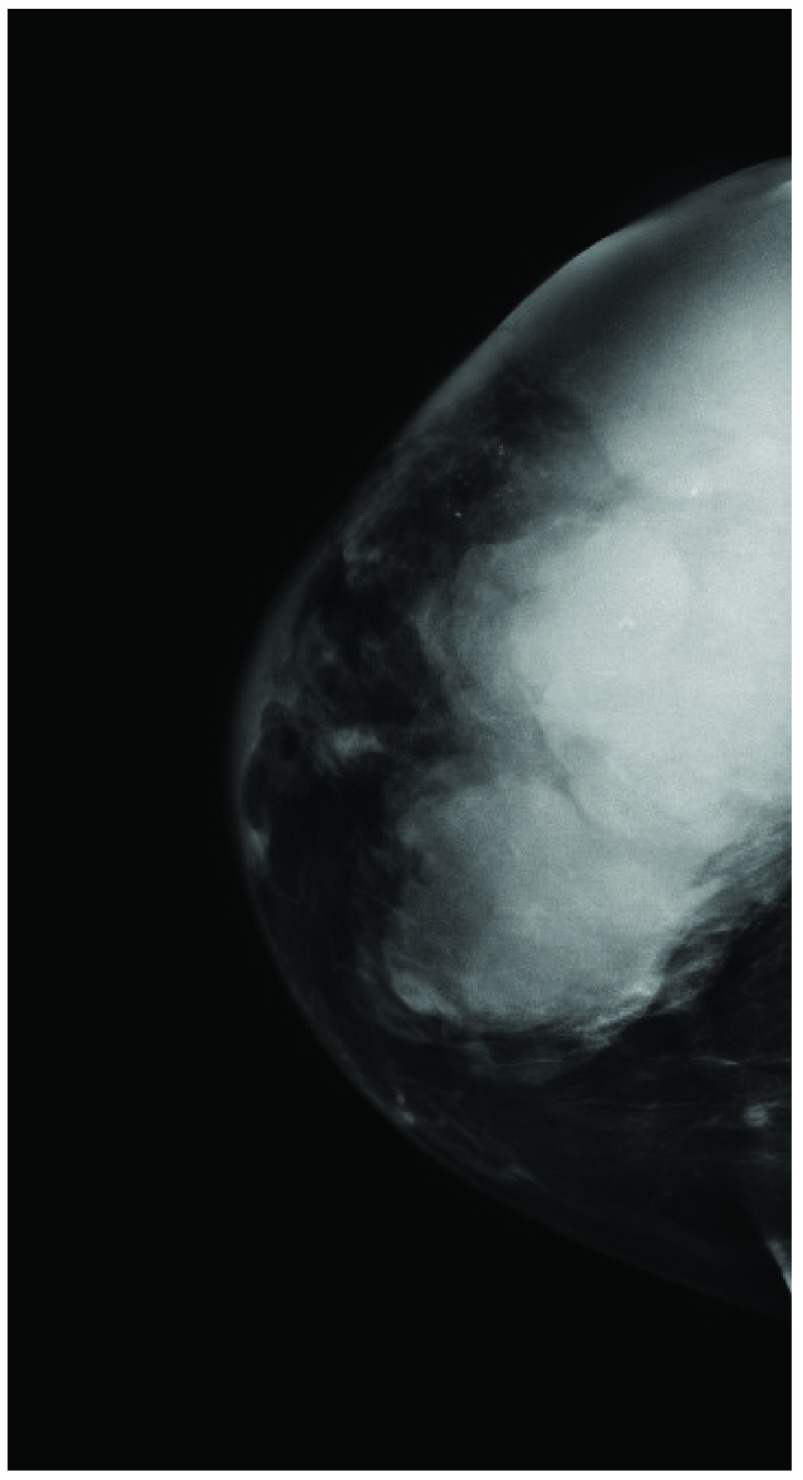
Digital mammography in the CC projection of the right breast demonstrates a large mass involving most of the right breast. There are scattered calcifications within the mass and overlying skin thickening. This corresponds to the visible and palpable abnormality and is strongly suspicious of malignancy (BIRADS 5).

**Figure 2.**
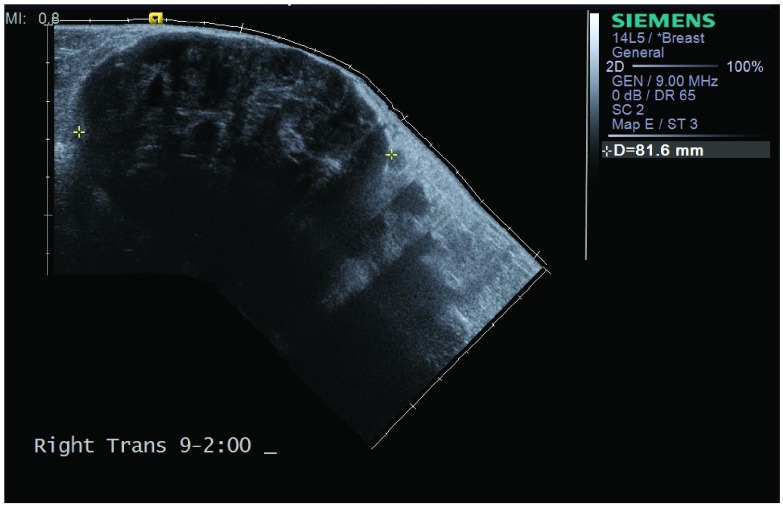
Right breast ultrasound demonstrates a large heterogeneous primarily hypoechoic lobulated mass encompassing most of the right breast.

**Figure 3.**
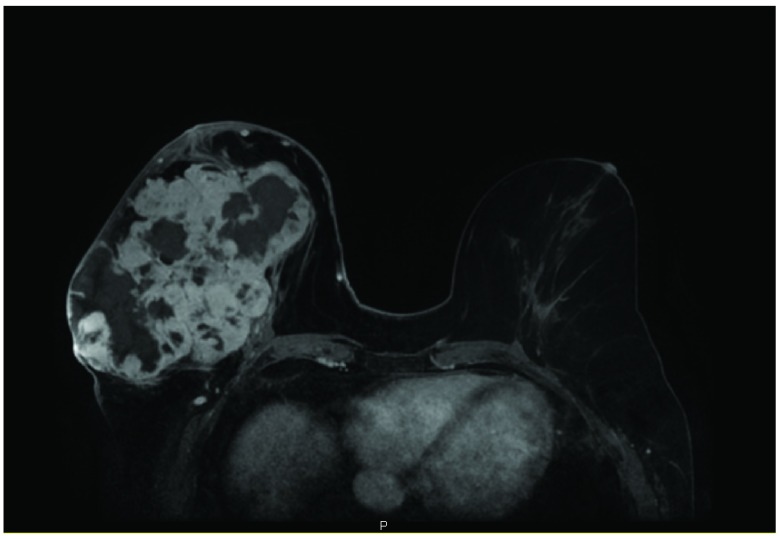
Breast MRI with contrast, axial view. In the right breast, there is a large lobulated heterogeneously enchancing mass with multiple areas of non-enchancement consistent with extensive necrosis. On axial image, the mass measures 15 cm × 9 cm × 13 cm. In the left breast, there is no suspicious mass. No suspicious adenopathy is seen in the axillae bilaterally.

A 58 year-old woman (G4P2) with no prior mammograms presented with complaint of increasing pain in her right breast for 7 months. Physical examination revealed an enlarged breast with multiple visible nodules but no adenopathy. Mammography detected a large mass associated with calcifications and thickening of the overlying skin (BIRADS 5) ( Figure 1). The left breast was normal. Sonographically, the mass was primarily hypoechoic ( Figure 2). MRI with contrast showed a lobulated, heterogeneously enhancing mass involving most of the right breast with multiple areas of necrosis. No lymphadenopathy or chest wall involvement was seen ( Figure 3).

Ultrasound guided core biopsy of the right breast revealed a spindle-cell neoplasm composed of tumor cells with blunt ended nuclei that were strongly positive for smooth muscle actin (SMA) and lacked expression of pan-cytokeratin, CD34, and S-100 (not shown). This immunophenotype is most consistent with a diagnosis of breast sarcoma. Metastatic workup detected small bilateral lung nodules.

In 2011 the patient underwent right total mastectomy with partial resection of the pectoralis muscle without chemo- or radiation therapy. Gross examination of the mastectomy specimen revealed a large (15 cm), firm, well-circumscribed mass. Microscopically, the tumor was composed of relatively bland spindle cells arranged as intersecting fascicles. The tumor was positive for SMA and vimentin, and negative for desmin, S-100, CD34, pan-cytokeratin, and neuron-specific enolase. A diagnosis of leiomyosarcoma was made. The resection margins were clean (> 1 cm).

**Figure 4.**
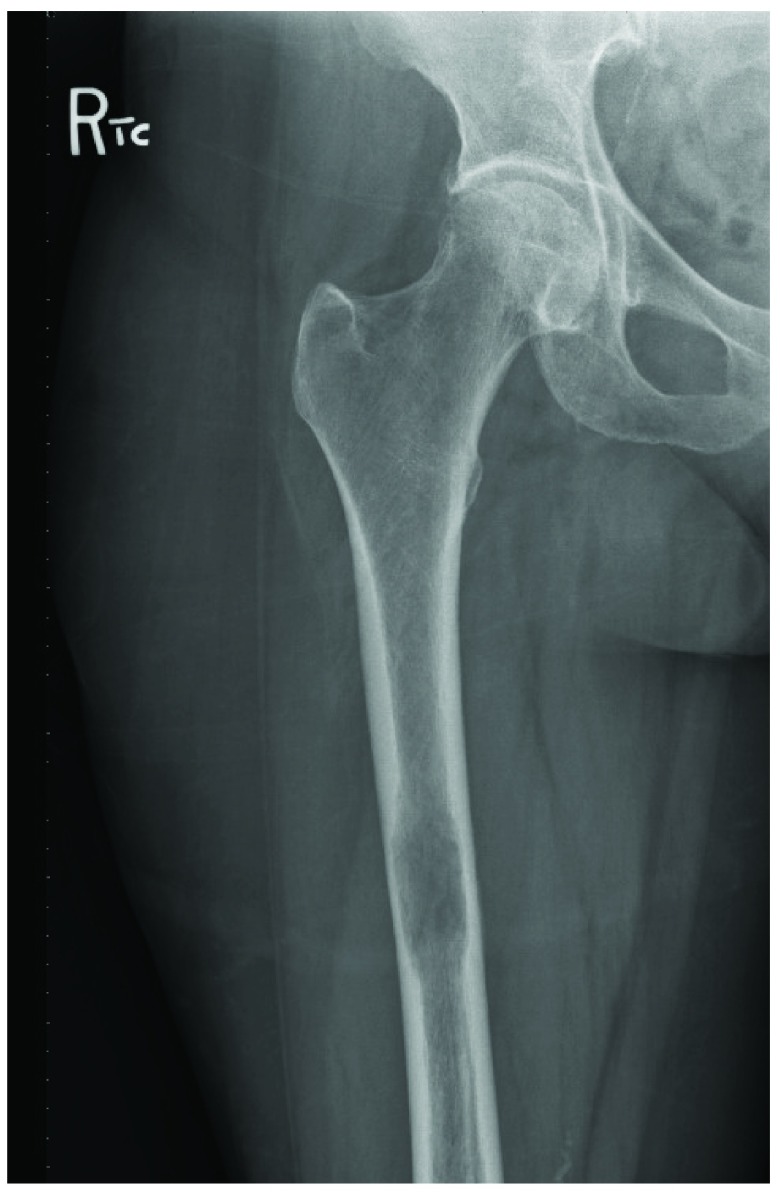
X-Ray right femur, AP view, demonstrates a large lucent lesion of the right femur with endosteal scalloping.

**Figure 5.**
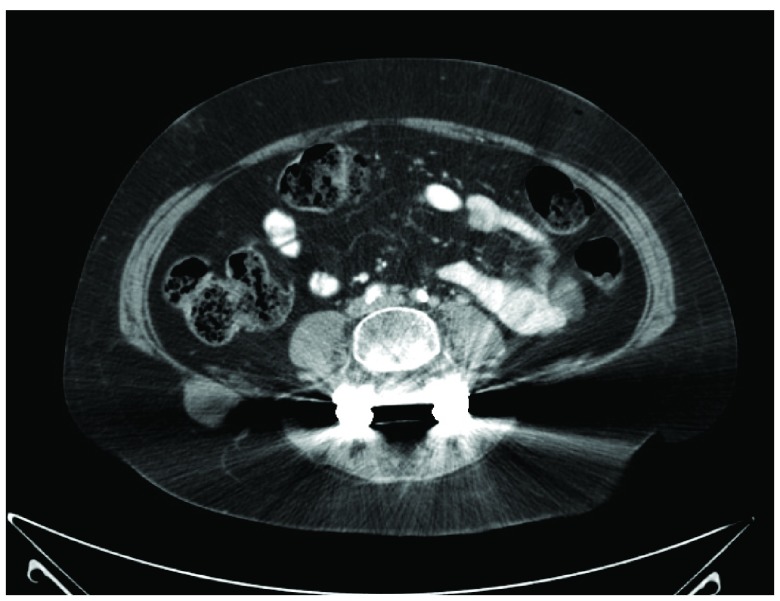
CT chest, abdomen and pelvis with contrast: A new 3 cm soft tissue mass is identified in the subcutaneous fat adjacent to the right iliac crest.

**Figure 6.**
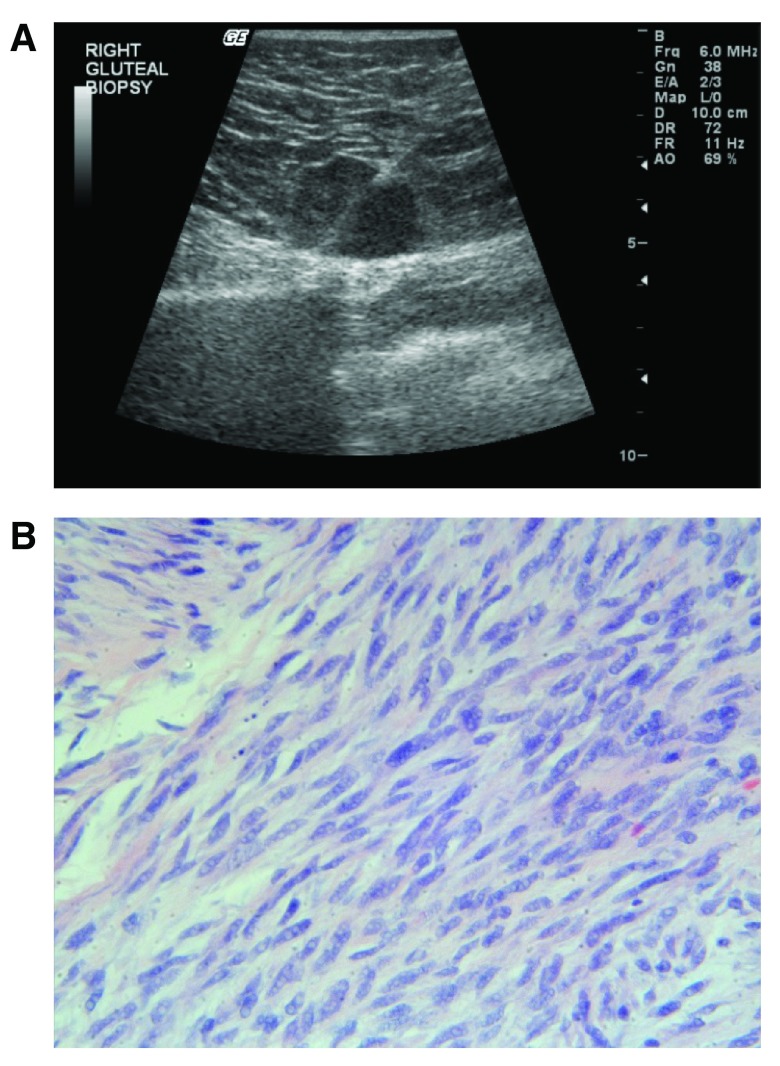
**A**. US guided right gluteal mass full-core biopsy with an 18-gauge BioPince. **B**. Metastatic neoplastic spindle shaped cells in the right gluteal lesion (H&E, 400X).

Two years later, the patient returned with a deep aching pain in her right knee and lower thigh. An X-Ray of her right femur showed a large lucent lesion with endosteal scalloping, suspicious for metastatic disease ( Figure 4). A repeat nuclear bone scan was positive for a new increased radiotracer uptake in the right femur. A CT of the chest, abdomen and pelvis discovered a new 3 cm soft tissue mass within the soft tissues in the right gluteal region and multiple lung nodules that were either new or have increased in size compared to previous CTs ( Figure 5). An ultrasound guided right gluteal mass full-core biopsy revealed a spindle cell neoplasm similar to the previously excised breast leiomyosarcoma, confirming the diagnosis of metastatic disease ( Figure 6). The metastatic gluteal and femoral tumors were resected and chemotherapy with Gemzar (gemcitabine, Eli Lilly) and Taxotere (docetaxel, Sanofi-Aventis) in a 21-day cycle was initiated. Despite chemotherapy, the lung nodules have been increasing in number and size (noted 2 months after the start of the chemotherapy).

## Discussion

Primary leiomyosarcoma of the breast is an extremely rare malignant neoplasm of uncertain biological behavior. There are less than 40 well-documented cases reported in the English medical literature ^1^. The majority of these cases presented as a well-circumscribed mass in the breast of postmenopausal women, although it has also been described in adolescent girls. The histogenesis of the entity is not clear. The myofibroblasts in the nipple areola complex have been proposed as the origin for the neoplasm ^2^. Most reported cases were relatively indolent but aggressive behavior with local recurrence and distant hematogenous metastasis to lungs and liver is also well-documented ^3^. The mainstay treatment is wide margin local excision. Most reported cases have undergone mastectomy with a few exceptions being treated with lumpectomy ^3^. Axillary dissection is believed to be unnecessary as the primary leiomyosaroma of the breast does not spread through the lymphatic route.

With a size of 15 cm, the present case represents the third largest tumor of all documented cases. Although bone is a common metastatic site for breast carcinoma, to the best of our knowledge breast leiomyosarcoma metastatic to the bone has not been reported. Prognostic factors predicting aggressive biological behavior in mammary leiomysarcomas are yet to be established ^4, 5^.

## Consent

Written informed consent for publication of clinical details and clinical images was obtained from the patient.
