# Supplementary material for: Case Report: Primary Leiomyosarcoma of the breast with unusual metastasis to the femur
Source: F1000Res. 2014 Sep 4;3:211. [Version 1] doi: 10.12688/f1000research.5213.1 (PMC4240243; doi:10.12688/f1000research.5213.1)
Supplement: Peer review history

# Peer review: Referee response for version 1

**DOI:** 10.5256/f1000research.5559.r6425

**Published:** 2014-11-17

**Authors:** Zsolt Baranyai

**Affiliations:** 1 Department of Surgery and Vascular Surgery, Uzsoki Memorial Hospital, Budapest, Hungary

The title covers the content and the message of the manuscript. The abstract summarizes the content of the article briefly and in a way easy to understand. The abstract is well-constructed and clear. The conclusion is sensible and well-balanced. Although the authors present a case first reported in the literature the conclusion is modest. There are no bias or competing interests.

The manuscript meets the criteria for case reports of the journal.

It reports the first diagnosed case of bone metastasis of a rare disorder, the leiomyosarcoma of the breast in a well-documented and straightforward way. Reviewing the literature no English article about a case like this was found, thus, the primary rank can be confirmed. The article is valuable not only due to the description of an extremely rare case, but it also calls the attention  to the possibility of bone metastasis in this disorder that should be sought for in the future in leiomyosarcoma. I fully support the indexing of the article.

I have read this submission. I believe that I have an appropriate level of expertise to confirm that it is of an acceptable scientific standard.

# Peer review: Referee response for version 1

**DOI:** 10.5256/f1000research.5559.r6424

**Published:** 2014-11-03

**Authors:** Offiong Ikpatt

**Affiliations:** 1 Department of Pathology, University of Miami, Miami, USA

The title, abstract and content are appropriate. No changes are required. For original research, the experimental design, including controls and methods, is adequate; results are presented accurately and the conclusions are justified and supported by the data.

I have read this submission. I believe that I have an appropriate level of expertise to confirm that it is of an acceptable scientific standard.
